# Evaluation of corneal hysteresis after pars plana vitrectomy combined phacoemulsification and intraocular lens implantation

**DOI:** 10.1038/s41598-022-18299-1

**Published:** 2022-08-26

**Authors:** Manami Ohta, Makiko Wakuta, Ayano Sakuma, Mina Hasegawa, Waka Hamada, Fumiaki Higashijima, Takuya Yoshimoto, Tadahiko Ogata, Yuka Kobayashi, Kazuhiro Kimura

**Affiliations:** 1 Department of Ophthalmology, Yamaguchi University Graduate School of Medicine, Ube, Yamaguchi 755-8505 Japan; 2 Clinical Research Center, Yamaguchi University Hospital, Ube, Yamaguchi 755-8505 Japan

**Keywords:** Diseases, Eye diseases

## Abstract

We evaluated the early effects of pars plana vitrectomy (PPV) on corneal biomechanics by comparing corneal hysteresis (CH) after cataract surgery (phacoemulsification and aspiration with intraocular lens implantation; PEA + IOL) alone and PPV combined with cataract surgery. This study included 20 eyes (18 patients), who underwent cataract surgery alone (PEA + IOL group), and 28 eyes (27 patients) who underwent PPV combined with cataract surgery (PPV triple group). The CH was 11.1 ± 1.1, 10.4 ± 1.1, and 11.0 ± 1.0 mmHg in the PEA + IOL group and 11.0 ± 1.4, 9.8 ± 1.4, and 10.6 ± 1.6 mmHg in the PPV triple group, preoperatively, at 2 weeks, and 3 months after surgery, respectively. The CH was not significantly different after surgery in the PEA + IOL group, but decreased significantly in the PPV triple group 2 weeks following surgery (p < 0.01). Intraocular pressure (IOP) and central corneal thickness (CCT) did not change significantly after surgery in either group. Preoperatively, there was a positive correlation between CH and CCT in the PPV triple group, but the correlation disappeared postoperatively. In PPV combined with cataract surgery, CH temporarily decreased postoperatively, independent of IOP and CCT. Removal of the vitreous may reduce the elasticity and rigidity of the entire eye.

## Introduction

Corneal hysteresis (CH) is an important feature that reflects the biomechanical properties of the cornea. Corneal biomechanics include all relevant factors such as intraocular pressure (IOP), corneal rigidity, and elasticity^1,2^. CH is affected by several factors, including aging, reduced central corneal thickness (CCT), high intraocular pressure (IOP), and long axial length (AL)^3–5^. Additionally, anterior chamber depth (ACD) is also believed to be a factor that impacts CH^6^. Moreover, there is a functional relationship between the anterior chamber and vitreous cavity because a positive correlation of IOP is shown between the two^7^. From a clinical perspective, CH has attracted attention for glaucomatous eyes. CH in glaucomatous eyes is lower than that in normal eyes and correlates with the severity and progression of visual field defects^8,9^. Decreased CH reflects deformation of the entire eye and is believed to be associated with weakness of the lamina cribrosa in glaucoma^10^.

Minimally invasive phacoemulsification and vitreous surgery have become widely used and are safely performed for various surgical indications^11–13^. However, some surgical complications may occur, and it is well known that elevated IOP can occur after vitrectomy^14^. In addition to the cornea, the sclera, lens, and vitreous are involved in ocular rigidity. The biomechanical properties of the cornea were significantly altered after cataract surgery^15^. Some reports have evaluated changes in CH by replacing the lens with an intraocular lens in cataract surgery^16–19^. The anterior chamber and vitreous cavity are also related to IOP maintenance^7^. The vitreous is a mechanical damper of the eye that absorbs shock^20^. The vitreous is involved in the elasticity and rigidity of the entire eye and contributes to maintenance of the eye structure. Indeed, vitrectomy alters the ACD in patients with retinal detachment and induces corneal remodeling and changes in CCT^21,22^.

The elastic properties of the cornea can be considered to reflect the elasticity of the entire eye, and the biomechanics of the cornea can be an indicator of the biomechanical properties of the entire eye^23^. Therefore, measuring CH enables estimation of ocular elasticity and rigidity^24^. Vitrectomy is also thought to affect the elasticity of the eye, but the impact of vitrectomy on CH remains unclear. In this study, we examined the effect of the vitreous on CH by comparing postoperative changes in CH between cataract surgery alone and vitrectomy groups. Additionally, we evaluated the correlation between CH and CCT before and after surgery in each group.

## Results

The patient demographics are summarized in Table 1. A total of 48 eyes (45 patients) were enrolled in this study: 20 eyes (18 patients) underwent only cataract surgery (PEA + IOL group), and 28 eyes (27 patients) underwent PPV combined with PEA + IOL (PPV triple group). There was a statistically significant difference in the mean ages of the PEA + IOL (74.8 ± 5.2 years), and PPV triple (69.9 ± 5.2 years) groups; p = 0.005. The causative diseases in the PPV triple group were epiretinal membrane (ERM) in 11 eyes and macular hole (MH) in 17 eyes combined with intravitreal sulfur hexafluoride (SF6) gas tamponade. Preoperative CCT, IOP, CH, ACD, and AL were not significantly different between the two groups.

**Table1:** Demographics of the study population.

Parameter	PEA + IOL group	PPV triple group	P value
Number (eyes)	20	28	–
Age ± SD(years)	74.8 ± 5.2	69.9 ± 5.2	0.005
Sex (male/female)	10/10	11/17	0.434
Diagnosis (eyes)	Cataract (20)	ERM (11) MH (17)	–
Surgical procedure (eyes)	PEA + IOL (20)	PEA + IOL + PPV + ILM peeling (11) PEA + IOL + PPV + ILM peeling + SF6 (17)	–
CH ± SD(mmHg)	11.0 ± 1.1	10.9 ± 1.5	0.676
IOPg ± SD (mmHg)	12.0 ± 2.8	13.2 ± 2.4	0.161
IOPcc ± SD (mmHg)	12.3 ± 2.6	13.5 ± 2.6	0.221
CCT ± SD (μm)	530.1 ± 35.9	541.5 ± 32.2	0.254
ACD ± SD (mm)	2.7 ± 0.3	2.5 ± 0.3	0.601
AL ± SD (mm)	23.5 ± 0.9	23.7 ± 1.2	0.107

*SD* standard deviation, *ERM* epiretinal membrane, *MH* macular hole, *PEA* phacoemulsification and aspiration, *IOL* intraocular lens, *PPV* pars plana vitrectomy, *ILM* inner limiting membrane, *SF6* sulfur hexafluoride, *CH* corneal hysteresis, *IOPg* Goldmann-correlated intraocular pressure, *IOPcc* corneal compensated intraocular pressure, *CCT* central corneal thickness, *ACD* anterior chamber depth, *AL* axial length.

The CH was 11.1 ± 1.1, 10.4 ± 1.1, and 11.0 ± 1.0 mmHg in the PEA + IOL group and 11.0 ± 1.4, 9.8 ± 1.4, and 10.6 ± 1.6 mmHg in the PPV triple group, preoperatively, 2 weeks, and 3 months after surgery, respectively. In the PEA + IOL group, there were no statistically significant differences in CH at any of the pre- or postoperative time points. In contrast, CH in the PPV triple group (p < 0.01) decreased significantly 2 weeks after the operation. In the PPV triple group, there was no difference in CH regarding the presence or absence of SF6 gas tamponade and causative diseases. The CH for eyes with SF6 gas tamponade were 10.81 ± 1.36, 9.96 ± 1.43, and 10.77 ± 1.36 mmHg, whereas for eyes without SF6 gas were 11.07 ± 1.62, 9.55 ± 1.23, and 10.36 ± 1.81 mmHg, preoperatively, and at 2 weeks, and 3 months after surgery, respectively. Consequently, there were no significant differences in CH with or without tamponade at any of the measurement points (preoperative: p = 0.48; post 2 weeks: p = 0.65; post 3 months: p = 0.79).

**Figure 1:**
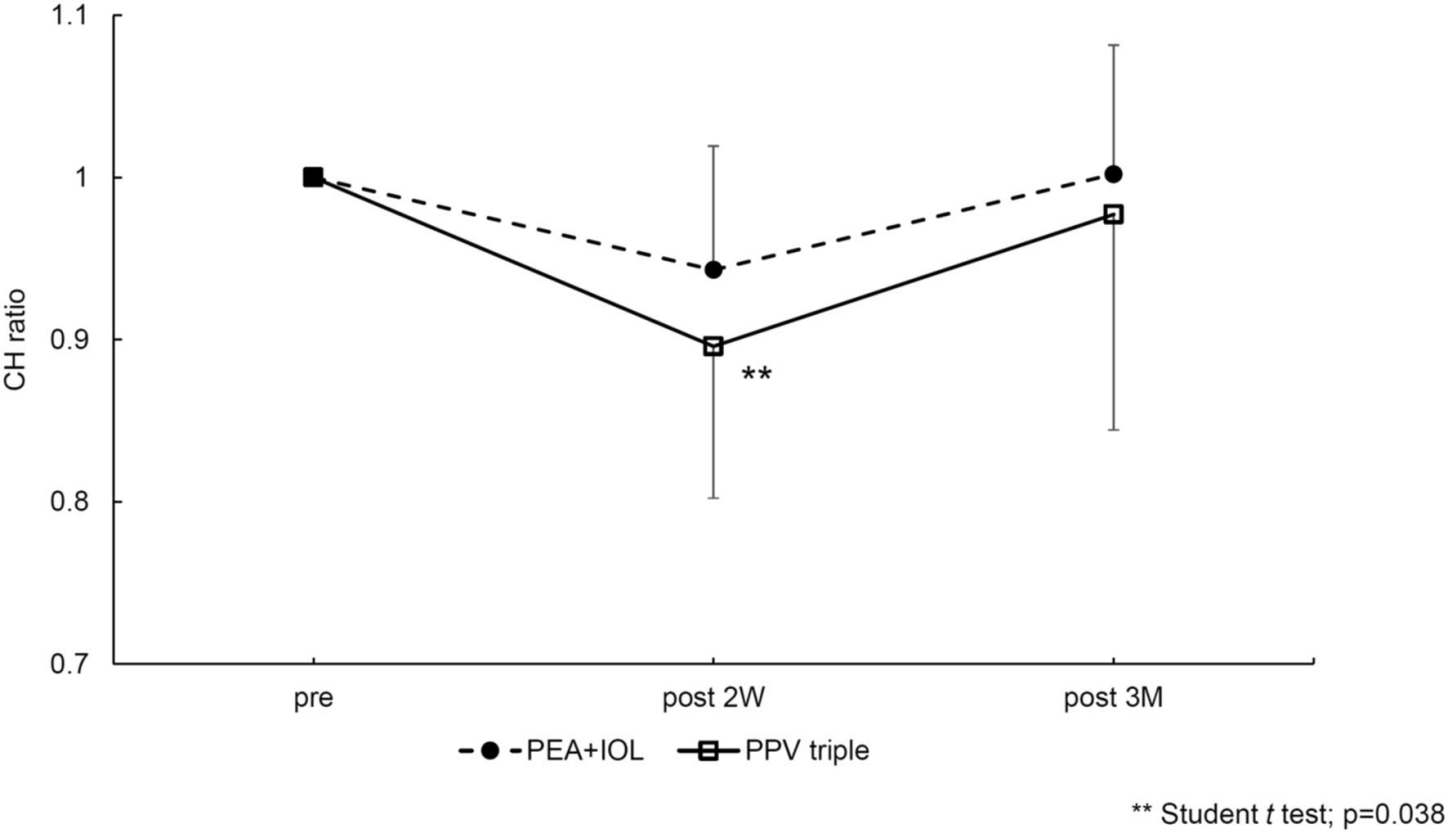
The postoperative to preoperative CH ratio of the PEA + IOL group was 0.94 ± 0.08, 1.00 ± 0.08, and for the PPV triple groups was 0.89 ± 0.09, 0.98 ± 0.13, 2 weeks, and 3 months postoperatively, respectively. The CH ratio was significantly smaller in the PPV triple group compared to the PEA + IOL group at 2 weeks postoperatively (**p < 0.05).

The postoperative to preoperative CH ratio of the PEA + IOL group was 0.94 ± 0.08, 1.00 ± 0.08, and for the PPV triple group was 0.89 ± 0.09, 0.98 ± 0.13, at 2 weeks, and 3 months after surgery, respectively (Fig. 1). In the between-group comparison, the CH ratio at 2 weeks after surgery was significantly lower in the PPV triple group than in the PEA + IOL group (p < 0.05).

**Figure 2:**
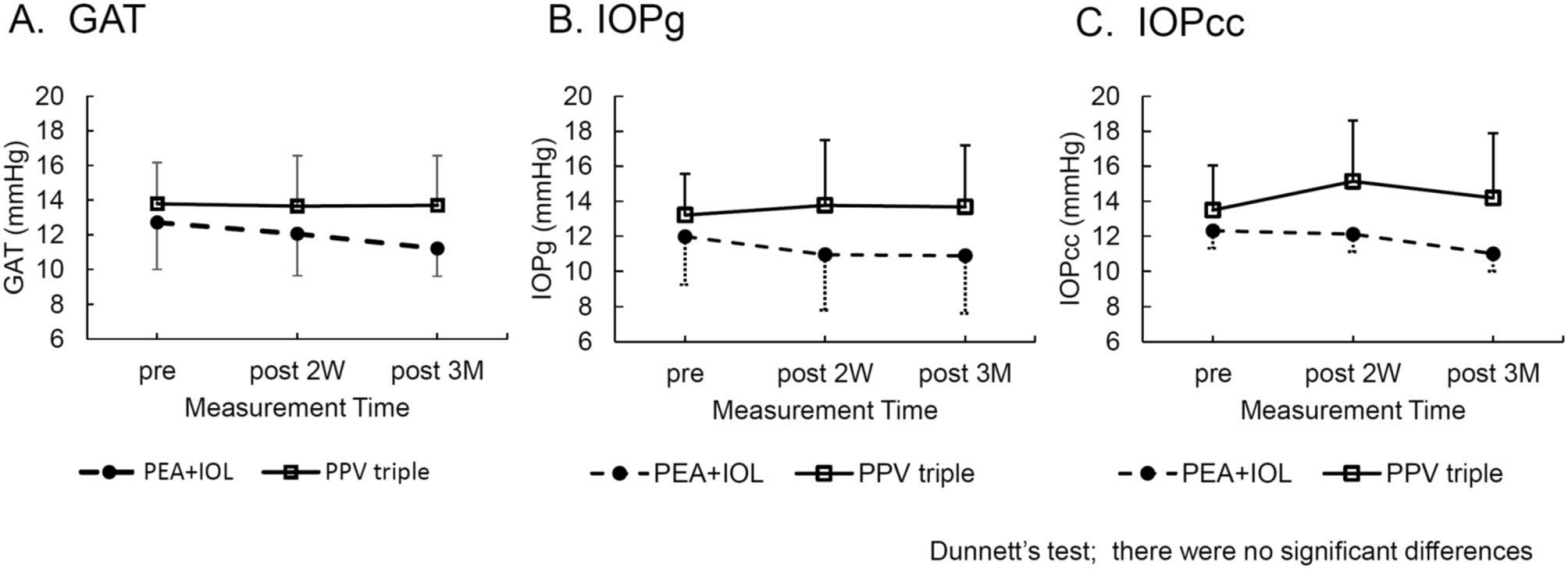
The Goldmann applanation tonometry (GAT) (**A**) (preoperatively, 2 weeks, and 3 months after surgery) of the PEA + IOL group was 12.7 ± 2.7, 12.1 ± 2.4 and 11.2 ± 1.6 mmHg, and PPV triple group was 13.8 ± 2.4, 13.7 ± 2.9 and 13.7 ± 2.9 mmHg. The IOPcc (**B**) of PEA + IOL group was 12.3 ± 2.6, 12.1 ± 2.6, and 11.0 ± 1.1 mmHg, and PPV triple group was 13.5 ± 2.6, 15.1 ± 3.4, and 14.2 ± 3.7 mmHg preoperatively, 2 weeks, and 3 months after surgery. The IOPg (**C**) PEA + IOL group was 12.0 ± 2.8, 11.0 ± 3.1, and 10.9 ± 3.3 mmHg, PPV triple group was 13.2 ± 2.4, 13.8 ± 3.7, and 13.7 ± 3.5 mmHg, preoperatively, 2 weeks, and 3 months after surgery, respectively. There was no significant difference in postoperative compared to preoperative IOP in both groups (p > 0.05).

The time course of IOP is shown in Fig. 2. The Goldmann applanation tonometry (GAT) (preoperatively, 2 weeks, and 3 months after surgery) of the PEA + IOL group was 12.7 ± 2.7, 12.1 ± 2.4, and 11.2 ± 1.6 mmHg, and that of the PPV triple group was 13.8 ± 2.4, 13.7 ± 2.9 and 13.7 ± 2.9 mmHg. The corneal compensated IOP (IOPcc) of the PEA + IOL group was 12.3 ± 2.6, 12.1 ± 2.6 and 11.0 ± 1.1 mmHg, and for the PPV triple group was 13.5 ± 2.6, 15.1 ± 3.4 and 14.2 ± 3.7 mmHg, preoperatively, 2 weeks, and 3 months after surgery. The Goldmann-correlated IOP (IOPg) of the PEA + IOL group was 12.0 ± 2.8, 11.0 ± 3.1 and 10.9 ± 3.3 mmHg, and that of the PPV triple group was 13.2 ± 2.4, 13.8 ± 3.7 and 13.7 ± 3.5 mmHg, preoperatively, 2 weeks, and 3 months after surgery, respectively. There were no significant differences in IOP between preoperative and postoperative values in either the PEA + IOL group (GAT: p = 0.14, IOPcc: p = 0.17, IOPg: p = 0.47) or PPV triple group (GAT: p = 0.98, IOPcc: p = 0.19, IOPg: p = 0.79).

**Figure 3:**
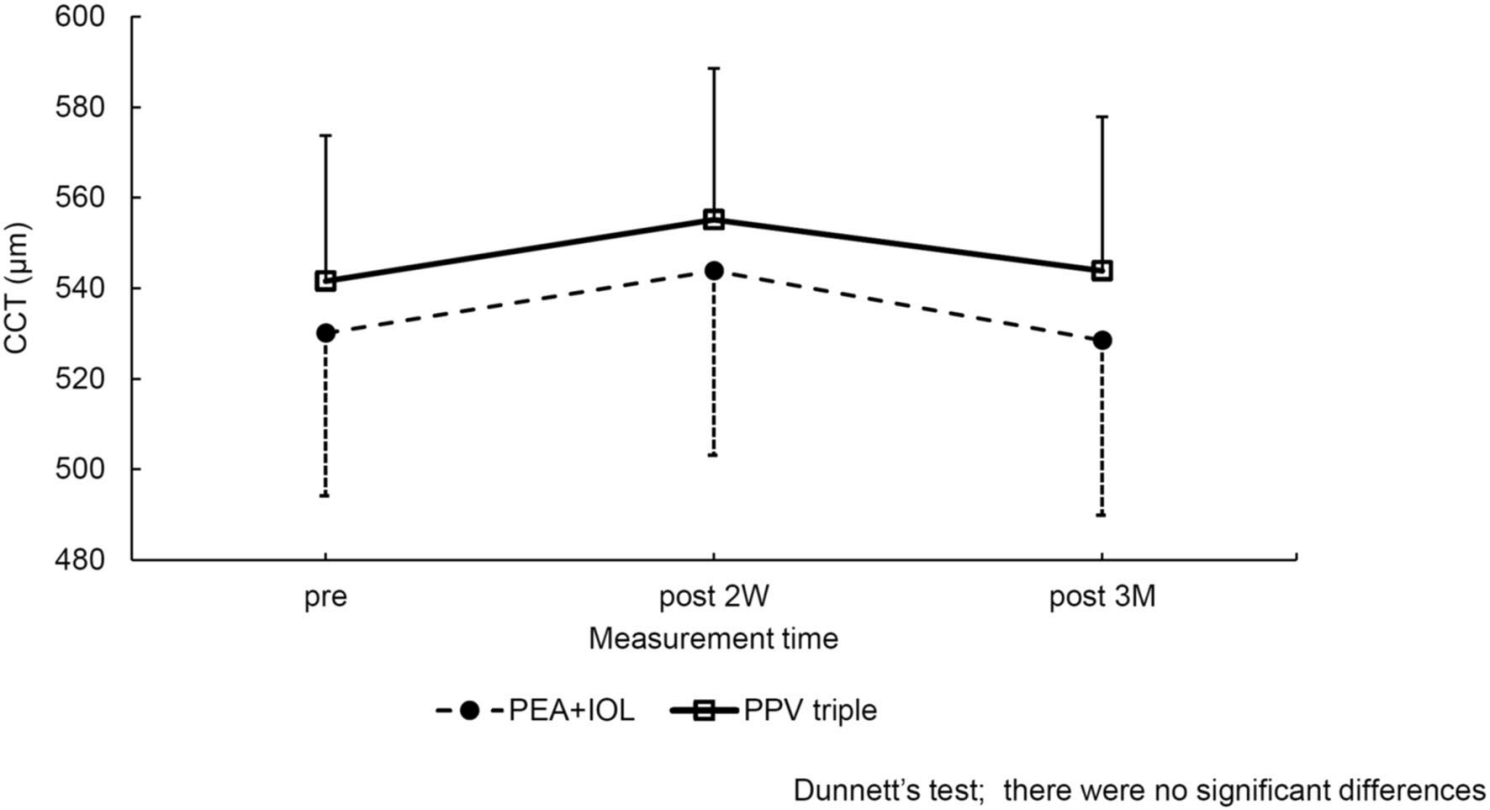
The CCT of the PEA + IOL group was 530.1 ± 35.9, 543.8 ± 40.7, and 528.5 ± 38.6 μm, the PPV triple group was 541.5 ± 32.2, 555.1 ± 33.5, and 543.8 ± 34.1 μm, preoperatively, 2 weeks, and 3 months after surgery, respectively. There was no significant difference in postoperative compared to preoperative CCT in both groups (p > 0.05).

The time course of CCT of the PEA + IOL group was 530.1 ± 35.9, 543.8 ± 40.7, and 528.5 ± 38.6 μm, and for the PPV triple group was 541.5 ± 32.2, 555.1 ± 33.5, and 543.8 ± 34.1 μm, preoperatively, 2 weeks, and 3 months after surgery, respectively. There were no significant differences between the preoperative and postoperative measurements in either the PEA + IOL group (p = 0.41) or PPV triple group (p = 0.28) (Fig. 3).

**Figure 4:**
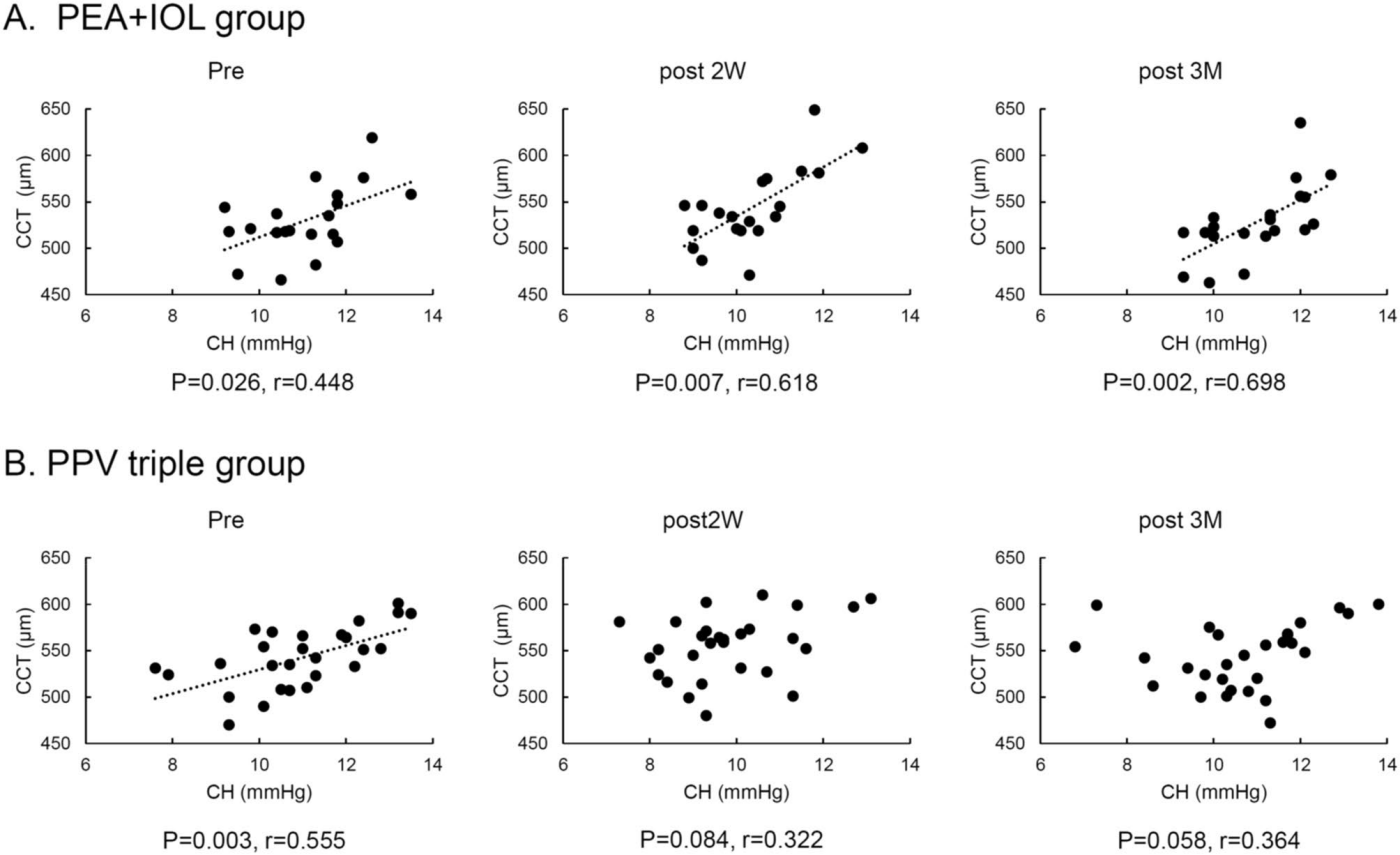
In PEA + IOL group (**A**), positive correlation between CH and CCT at all-time points; r = 0.448, p = 0.026 preoperatively, r = 0.618, p = 0.007 at 2 weeks postoperatively, r = 0.698, p = 0.002 at 3 months postoperatively. In the PPV triple group (**B**), positive correlation between CH and CCT preoperatively (r = 0.555, p = 0.003), but there was no correlation after surgery; r = 0.322, p = 0.084 at 2 weeks postoperatively, r = 0.364, p = 0.058 at 3 months postoperatively.

The correlation between CH and CCT in the PEA + IOL and PPV triple groups is depicted in Fig. 4. Significant correlations were observed in the PEA + IOL group before surgery (r = 0.448; p = 0.026), or at 2 weeks (r = 0.618; p = 0.007), or 3 months (r = 0.698; p = 0.002) after surgery. In the PPV triple group, there were significant correlations preoperatively (r = 0.555; p = 0.003), which disappeared at 2 weeks (r = 0.322; p = 0.084) and 3 months (r = 0.364; p = 0.058), after surgery.

The correlation between preoperative ACD, AL, and CH was not statistically significant at all measurement points for both the PEA + IOL and PPV triple groups (data not shown).

## Discussion

In this study, we measured CH in the PEA + IOL and PPV triple groups to investigate the involvement of the vitreous, a mechanical damper that maintains elasticity and rigidity of the entire eye. Changes in CH were measured in the PEA + IOL and PPV triple groups preoperatively and at 2 weeks and 3 months after surgery. In the PEA + IOL group, there were no statistically significant differences in CH at any time point. However, in the PPV triple group, there was a significant decrease in CH at 2 weeks postoperatively.

We demonstrated that CH was significantly reduced at 2 weeks following 25-gauge vitrectomy. In this study, we compared the PEA + IOL group with the PPV triple group; the difference between the two groups was in the creation of scleral wounds and the removal of the vitreous, and there were no significant differences in surgical invasion to the cornea. In addition, there were no differences in parameters such as preoperative CH between two groups, and IOP did not change significantly pre- and post-operatively, and CCT was not associated with CH at any time point after vitrectomy. CH is a measure of the elasticity or rigidity of the cornea and is not a direct measurement of the entire eye. However, based on these findings, we hypothesized that the postoperative CH changes observed in the PPV triple group were due to the creation of a scleral wound and the removal of the vitreous, which may have affected the elasticity or fragility of the entire eye, which resulted in CH changes. Whether CH reflects the elasticity and rigidity of the entire eyeball is not clear, and further study is warranted.

Several previous reports have described the impacts of PPV on CH. Seymenoğlu et al.^25^ reported a significant decrease in CH 1 month after 23-gauge PPV, and CH was correlated with an increase in CCT preoperatively and at 1 and 3 months postoperatively. These results reveal that microincision vitreous surgery temporally decreases CH early after surgery, and removal of the vitreous by vitrectomy might have affected the elasticity and rigidity of the entire eye. For 3 months after 23-gauge vitrectomy, CH was correlated with elevated CCT. In this study, CH was not correlated with elevated CCT after 25-gauge vitrectomy. Previous reports revealed that both vitrectomy gauges are equally effective in treating vitreoretinal diseases regarding clinical outcomes and complications^26^. However, corneal remodeling and CCT changes after 23-gauge vitrectomy are sometimes detected^22^. These results suggest that the selection of a small gauge size may influence the elasticity and rigidity of the entire eye through the corneal structure.

There are some reports on the changes in corneal biomechanical parameters after cataract surgery. It is also known that CH decreases transiently in the early postoperative period but subsequently recovers to preoperative levels. Hager et al.^16^ reported on 101 eyes that underwent PEA and IOL implantation through a 4.1 mm corneal incision, in which CH decreased and CCT increased on the first postoperative day. Kamiya et al.^17^ reported on 54 eyes that underwent PEA and IOL implantation through a 2.8 mm corneal incision; CH significantly decreased and CCT increased 1 day postoperatively, and subsequently recovered to preoperative levels at 1 week after surgery. They speculated that temporary corneal edema caused by acute surgical stress on the corneal endothelium reduces the energy absorption capacity of the corneal tissue. In the present study, there was no significant difference in the preoperative and postoperative CH in the PEA + IOL group; the CH may have already recovered at 2 weeks postoperatively due to the recovery of corneal edema. We observed a decrease in CH in the PPV triple group and no change in CCT 2 weeks after surgery. These results suggest that anterior surgical inflammation in PEA + IOL surgery was not related to a decrease in CH at 2 weeks after PPV triple surgery.

In this study, CH decreased significantly at 2 weeks postoperatively in the PPV triple group, and the preoperative to postoperative CH ratio was significantly lower than that in the PEA + IOL group. It took longer for CH to recover to preoperative levels in the PPV triple group. CH was negatively correlated with IOP and ACD and positively correlated with CCT and peri-temporal corneal thickness^27^. Furthermore, CH is influenced by several parameters, including corneal elasticity and the ocular axis^3,5,28^. In addition, transient IOP elevations after PPV and cataract surgery are well known and can be caused by inflammation, residual viscoelastic substances, steroid responders, and intraocular tamponades, such as gas and silicone oil^29–31^. In the present study, there was no significant difference in IOP between the groups in the preoperative and postoperative comparisons because we excluded patients with hypertension requiring postoperative ophthalmic treatment. Therefore, it is unlikely that the postoperative increase in IOP caused the decrease in CH. The CCT at 2 weeks postoperatively was not significantly different between the groups. The recovery of CH to preoperative levels at 3 months postoperatively is similar to that in a previous report^25^, but since there was no increase in postoperative CCT in this study, the effect of CCT is unlikely. Congdon et al.^8^ hypothesized that in glaucoma, CH reflects not only the cornea but also global ocular biomechanics. We also examined the relationship between ACD and CH but observed no significant correlation. Based on our previous results, vitreous removal may have influenced the transient decrease in CH at 2 weeks after vitreous triple surgery.

Our results also showed that the CH returned to preoperative levels 3 months postoperatively in avitreous eyes, while the cause of CH recovery in the late postoperative period is not clear. It has been reported that scleral healing by observed ultrasound biomicroscopy takes 4 weeks in 25-gauge vitrectomy^32^. Gozawa et al.^33^ reported postoperative histological changes in scarring after microincision vitrectomy surgery. At postoperative day 1 to 7, i infiltration of myofibroblasts and neutrophils in incision sites was observed, and subconjunctival tissues and sclera were ticker and recovered at postoperative 1 months. The time course of scleral wound healing after PPV in these reports was consistent with the postoperative CH changes in this study.

Metabolism of vitreous collagen in avitreous eyes after PPV may affect CH changes. Itakura et al.^34^ collected fluid samples after vitrectomy and compared these with vitreous samples, reporting that hyaluronan levels were significantly decreased, but the C-propeptide levels of type II procollagen were higher. It suggests that type II collagen may be persistently secreted in avitreous eyes. It was also reported that the ciliary body serves as a source of vitreous collagen^35^ and plays a role in the recovery function of mucopolysaccharide in the vitreous body. Therefore, the composition of the vitreous cavity changes even after vitrectomy, which may affect the reduction and recovery of CH after PPV. Tissue adaptations, such as wound healing of the sclera and changes in the composition of the avitreous body, may have contributed to the recovery of postoperative CH.

The limitations of this study were the limited amount of data and the small number of postoperative measurements: CH, CCT, etc. were assessed at 2 weeks and 3 months postoperatively, but larger cases with more measurement points and longer term studies are needed for further assessment of postoperative changes in CH. In addition, because this was a retrospective study, it was not possible to match the ages between the two groups, and the PEA + IOL group was significantly older. In this study, baseline CH did not differ between the two groups, but CH responsiveness after surgery may differ with age.

In PPV, CH decreased for a longer period following surgery. During this time, the rigidity and elasticity of the entire eye are reduced, and the eye structure is considered to be affected by external forces or ocular hypertension. CH was significantly lower in patients with high myopia than in healthy subjects^36,37^. In addition, the biomechanics of the cornea may reflect the biomechanics of the eye and predict the development of glaucoma^9^. Examining corneal biomechanics after vitrectomy can provide insight into corneal behavior under particular circumstances, and may also reflect vulnerability of the optic nerve head to glaucoma, and the severity of myopia.

## Methods

This was a single-center retrospective study. The procedures were approved by the Ethics Committee of Yamaguchi University Hospital and complied with the guidelines of the Declaration of Helsinki. Informed consent was obtained from all participants.

### Study population

All participants underwent cataract surgery (phacoemulsification and aspiration with intraocular lens implantation; PEA + IOL) or pars plana vitrectomy (PPV) combined with PEA + IOL for ERM or MH at Yamaguchi University Hospital between July 2018 and July 2021. Patients with glaucoma, corneal degeneration such as keratoconus, history of uveitis, history of ophthalmic surgery, high myopia of-6 D or more, and intraoperative complications (either PEA + IOL or PPV) were excluded. Patients with postoperative elevated IOP who required additional drug therapy were also excluded.

### Surgical procedures

Cataract surgery PEA + IOL was performed using standard surgical techniques, comprising continuous curvilinear capsulorrhexis, nuclear and cortex extraction with phacoemulsification and aspiration (PEA) with Whitestar Signature Pro (Johnson & Johnson Consumer Company, New Brunswick, New Jersey, USA), and intraocular lens (IOL) implantation through a 12 o’clock 2.65 mm scleral and corneal tunnel incision.

PPV was performed using the Constellation Vision System (Alcon Laboratories, Inc., Fort Worth, Texas, USA) with 25-gauge instrumentation and a wide-angle noncontact viewing system (Resight 500; Carl Zeiss Meditec AG, Oberkochen, Germany). Four ports were created 3–4 mm posterior to the limbus. PPV consisted of core vitrectomy and peripheral vitrectomy with triamcinolone acetonide (MaQaid, Wakamoto Pharmaceutical Co., Ltd., Tokyo, Japan) to visualize the vitreous gel and for inner limiting membrane peeling. Patients with macular holes underwent fluid-air exchange and 1.5 ml of 100% SF6 gas was injected through the pars plana at the end of surgery. The 25-gauge scleral ports were not sutured. Patients with macular holes were kept in a prone position for 3 days to 1 week after surgery. The patients used steroidal, antibiotic, and diclofenac sodium eye drops for three months postoperatively.

### Measurements of ocular biomechanical parameters

Corneal hysteresis was measured using an Ocular Response Analyzer (ORA; Reichert Ophthalmic Instruments, Depew, NY, USA) before, 2 weeks, and 3 months after surgery. In ocular response analysis, the cornea is compressed until it becomes concave, and then decompressed until it becomes flattened by a rapid air pulse. The time required for the cornea to flatten inward and outward was measured and the air pressure at these two points was calculated. The pressure difference between inward (P1) and outward (P2) flattening processes occurs due to the influence of corneal biomechanical properties, and this difference (P1–P2) is defined as CH^24^. The ORA also determined two IOP measurements: Goldmann-correlated IOP (IOPg), which is the average of pressure P1 and P2 and is equivalent to the Goldmann applanation tonometer measurement, and corneal compensated IOP (IOPcc), which is less affected by corneal thickness and is intended by the manufacturer. These values were measured once at each time and measurements were adopted using a waveform score of 7 or higher, which represents reliability. IOP was also measured by a physician using Goldmann applanation tonometry (GAT).

CCT and ACD were measured using the CASIA SS-1000 (TOMEY, Nagoya, Japan), and ALs were measured using the IOL Master 500 (Carl Zeiss Meditec, Dublin, CA).

### Statistical analysis

Student’s *t*-test for parametric variables and Mann–Whitney U test and chi-squared test for nonparametric variables were used to determine between-group differences. The multiple comparison test for parametric variables was performed using the Dunnett test to determine whether the postoperative CH changed from that preoperatively, as a control. Pearson’s correlation coefficient test was used to determine the significance of the correlation coefficients between CH and CCT, ACD, and AL. All statistical analyses were performed using the Statcel 4 software (OMS Publishing Inc. Saitama, Japan), and statistical significance was set at p < 0.05.

## Data Availability

The datasets generated and analyzed during the current study are available from the corresponding author on reasonable request.
